# Relational Stability in the Expression of Normality, Variation, and Control of Thyroid Function

**DOI:** 10.3389/fendo.2016.00142

**Published:** 2016-11-07

**Authors:** Rudolf Hoermann, John E. M. Midgley, Rolf Larisch, Johannes W. Dietrich

**Affiliations:** ^1^Department of Nuclear Medicine, Klinikum Luedenscheid, Luedenscheid, Germany; ^2^North Lakes Clinical, Ilkley, UK; ^3^Medical Department I, Endocrinology and Diabetology, Bergmannsheil University Hospitals, Ruhr University of Bochum, Bochum, Germany; ^4^Ruhr Center for Rare Diseases (CeSER), Ruhr University of Bochum, Bochum, Germany; ^5^Ruhr Center for Rare Diseases (CeSER), Witten/Herdecke University, Bochum, Germany

**Keywords:** TSH, thyroid hormones, TSH feedback control, thyroid homeostasis, set point

## Abstract

Thyroid hormone concentrations only become sufficient to maintain a euthyroid state through appropriate stimulation by pituitary thyroid-stimulating hormone (TSH). In such a dynamic system under constant high pressure, guarding against overstimulation becomes vital. Therefore, several defensive mechanisms protect against accidental overstimulation, such as plasma protein binding, conversion of T4 into the more active T3, active transmembrane transport, counter-regulatory activities of reverse T3 and thyronamines, and negative hypothalamic–pituitary–thyroid feedback control of TSH. TSH has gained a dominant but misguided role in interpreting thyroid function testing in assuming that its exceptional sensitivity thereby translates into superior diagnostic performance. However, TSH-dependent thyroid disease classification is heavily influenced by statistical analytic techniques such as uni- or multivariate-defined normality. This demands a separation of its conjoint roles as a sensitive screening test and accurate diagnostic tool. Homeostatic equilibria (set points) in healthy subjects are less variable and do not follow a pattern of random variation, rather indicating signs of early and progressive homeostatic control across the euthyroid range. In the event of imminent thyroid failure with a reduced FT4 output per unit TSH, conversion efficiency increases in order to maintain FT3 stability. In such situations, T3 stability takes priority over set point maintenance. This suggests a concept of relational stability. These findings have important implications for both TSH reference limits and treatment targets for patients on levothyroxine. The use of archival markers is proposed to facilitate the homeostatic interpretation of all parameters.

## Introduction

Maintenance of body composition and its internal milieu both over long periods of time and during varying imposed external conditions is a primary goal for attaining human health. This is expressed as biological normality, which is more narrowly defined as a perceived optimal state for an individual rather than through a reference range for a population (1–3). In contrast, the statistical definition of normality is inferred by the Gaussian distribution of the variation or measurement error frequently observed in a population (4). Biological variation is not expressed by an error term, but has an important evolutionary role (5).

The human body is essentially a highly complex, interactive biological system where normality and variation are tightly controlled by various mechanisms (6, 7). Typically, the hypothalamus–pituitary–thyroid–axis is closely concerned with energy metabolism and multiple specific functions within the organism. Gaining control over energy expenditure independently of environmental short-term supply offers major systemic advantages (8). It is therefore unsurprising that the thyroid, within the broader biological complex, embodies these basic biological necessities, showing also associations with longevity in older populations (9–13). By nature, such systems tend to operate far away from their unstimulated resting point, and being metastable require considerable energy and systemic pressure in order to avoid collapse and to maintain a desired equilibrium point. Subtle variations in responding to challenges either by an individual or among populations provide stabilization against adventitious reactions that could otherwise cause wide fluctuations and turbulences in the control outputs (14).

In this short review, we will examine the expression of normality, variation, and control of thyroid function in humans and draw some conclusions on their practical use for diagnosis and treatment.

## Background

The human thyroid gland produces and then releases into the circulation, a large amount of thyroxine (T4) and a lesser proportion of triiodothyronine (T3). Hormone concentrations in the circulation depend on both specific and unspecific binding to proteins, such as TBG, transthyretin, and albumin. Only very small concentrations exist as the respective unbound free forms. The free molecules are biologically active, free T3 (FT3) significantly more so than free T4 (FT4). The two hormones are interrelated by conversion of T4 into T3 by enzymatic 5′ monodeiodination (15, 16). Most of T4 circulates throughout the body, whereas T3 is predominantly found within cells. T3 transport in and out of the cell is potentiated *via* specific transport mechanisms and not by passive diffusion (17, 18). Within the cell, T3 is eventually transported to the nucleus where it binds to thyroid hormone receptors to exert its mostly genomic actions (19–21). Non-classical actions include binding of thyroid hormones to membrane receptors (22). The basal unstimulated glandular output of thyroid hormones is relatively low (23). It potentiates the euthyroid state only upon stimulation by pituitary thyroid-stimulating hormone (TSH). This puts the system of T4 and T3 production under constant high pressure. Consequently, such a high pressure system requires defensive mechanisms to protect the cells from the dangers of being flooded and overwhelmed by an over-supply of thyroid hormones. This includes binding of thyroid hormones to plasma proteins, prior activation of the pro-hormone T4 (T4–T3 conversion), gate control at cell entry (active transport across the cell membrane), and production of locally expressed counter-regulatory derivatives such as reverse T3 and thyronamines that exert short loop inhibitory control (17, 24–26). There is additionally a systemic negative feedback control at the pituitary and hypothalamic level that both controls thyroid hormone production and sets an appropriate internal reference point for pituitary TSH secretion (27).

Modern assays allow readily available measurements of all three thyroid parameters, TSH, FT4, and FT3 (28–30). This does not apply to TRH whose circulatory concentrations are too low for reliable detection and result from multiple sources including hypothalamus, spinal cord, and gastrointestinal tract. At the present time, there are 27,184 publications on TSH in PubMed. However, most studies have focused exclusively on TSH, or examined the parameters in isolation, downplaying the interrelationships with the other thyroid hormones. TSH has assumed a dominant and statistically independent role as the result of its exceptional sensitivity, compared to thyroid hormones, thus divorcing it from its physiological roots as an indirect controlling element (27, 29, 31, 32). This, in turn, has fostered a widely held belief that its exceptional sensitivity in response translates into superior diagnostic performance, thereby making the additional consideration of thyroid hormones largely redundant (33). We have examined the validity of this tenet below.

## Normality of Thyroid Parameters

Statistical normality applies to FT4 and FT3 in a sufficiently large and healthy population sample, thus making it easy to define appropriate 95% confidence limits or reference intervals for a population. For TSH, which is not normally distributed, a logarithmic transformation was used as an accepted statistical procedure (34). This method has had limited success and the remaining skewness of the logarithmically transformed TSH was explained by the putative presence of clinically hidden pathologies such as thyroid autoimmunity that are highly prevalent in the population (35–38). Further investigations showed considerable unexpected variation in the upper limit of the reference range (39–41). This hiatus in defining the TSH reference range has been widely discussed and various influences, among others, methodology, geography, ethnicity, and age have been suggested to explain the discrepancies (34–54). However, the disagreement has not been resolved. This may indicate an important underlying problem.

By its physiological role as discussed above, TSH is interlocked with FT4, being the main driving force behind the rise in concentration of the latter hormone to its normal euthyroid level. In homeostatic equilibrium, the two values deliver the so-called set point (55). This represents the intersecting point between the characteristic curves for thyroidal FT4 production and the pituitary response of TSH feedback control. The set point is less variable in an euthyroid individual, and, importantly, intraindividual variability of TSH is only about half as wide as its interindividual variability (55–59). TSH differs thereby from many other laboratory parameters where intra-subject and between-subject variation are nearly equal. A two-dimensional or three-dimensional distribution of TSH and FT4/FT3 in the euthyroid range describes clusters of set points appropriate for healthy individuals (60–64). Conceptionally, this questions the use of the presently employed isolated univariate reference ranges for single parameters and promotes a composite expression of multivariate normality in the collective. Between-subject variation should therefore best be addressed by paired measurements of TSH and FT4 in healthy individuals. Using a large sample from a prospective study, we have derived bivariate and trivariate reference limits for TSH and thyroid hormones (64). We have further examined their diagnostic performance, compared to a univariate TSH reference range (64). This study revealed frequent discrepancies in the placement of results between composite multivariate reference limits and a combination of the univariate single reference intervals. Method-associated reclassification from thyroid dysfunction to euthyroidism was as high as 26% by using the bivariate limit or 42% for the trivariate limit, respectively (64). These recent findings agree with the few previous studies applying the concept of multivariate normality to clinical data (60, 63), extending the earlier findings to evaluating diagnostic performance (64). This demonstrates that statistical analytic techniques heavily influence current TSH-reliant thyroid disease classification. Hence, joint application of the dual roles of TSH as a sensitive screening test and an accurate diagnostic tool becomes highly questionable and consequently the roles must be separated from each other. The current classification of the disease entities of subclinical hypothyroidism or hyperthyroidism, which is solely based on abnormal TSH values when thyroid hormones concentrations remain within their respective reference ranges, seems no longer tenable (31, 32). New markers for clinical endpoints or tissue-based definitions of thyroid function are therefore urgently needed. Over-reliance on TSH as a gold standard has long impeded the advancement of the field, since the first doubts were raised and disagreements emerged on the setting of the reference intervals (34–54). While sole reliance on TSH must therefore be scaled back, good clinical practice taking into account the full history and symptoms displayed by a patient has to be re-instituted as a primary tool (65, 66).

## Relational Stability between Thyroid Parameters

Although current definitions of subclinical thyroid dysfunction follow a narrow TSH-based distinction, recent studies demonstrated that the cardiovascular and mortality risk increases within the “normal” thyroid function range (67–69). This suggests that transition into thyroid disease occurs more gradually, and risks may not be reliably assessed by univariate TSH normality. The situation is further complicated by issues with TSH reference limits discussed above. A combined view of all thyroid parameters and their interrelationships may provide a more comprehensive picture as a moderately raised TSH could either indicate a failed attempt at restoration of euthyroidism or signal successful homeostatic adaptation. The expression of adaptive homeostatic equilibria between TSH and thyroid hormones may provide an early defense line against the abrupt onset of thyroid failure following less severe or temporary disruptions. Clinical studies support this view showing no adverse outcomes for mortality, cardiovascular events, fracture risk, or cognitive impairment in association with mildly elevated TSH levels (5–10 mIU/l) (70). In elderly patients with subclinical hypothyroidism, in contrast, life expectancy was found to be compromised with lower, not higher TSH values (9–13).

We hypothesized that early adaption under system stress should be testable by studying the expression of homeostatic equilibria across the spectrum of euthyroid subjects (71). The concept was termed relational stability, describing adaptive interrelations between the parameters rather than univariate expression of normality of a single component maintaining system stability. We found an inverse correlation between TSH-standardized T4 production and T4–T3 conversion across the euthyroid reference range (71). This contradicts the assumption that variation between thyroid hormones and TSH may be randomly defined in euthyroid subjects by genetic variation in the formation of set points (72–74), rather indicating early homeostatic control across the euthyroid spectrum (71). The euthyroid reference range for FT3 becomes dependent on a progressive alteration in the controlling interplay between TSH, FT4, and FT3 across the range. The concept extends to the diseased state, e.g., in patients with autoimmune thyroiditis where the observed pattern of control was similar, albeit shifted at a lower level (71).

Hence, maintaining stable FT3 positions takes priority over set point fixation in expressing the TSH–FT4–FT3 relationship, as the system seeks early compensation from the very onset of thyroid capacity stress, progressively increasing global deiodinase activity as thyroidal production declines (71). This T3-stabilizing behavior may emanate from the expression of TSH feedforward control on deiodinase activity (27). Phase-shifted coupling of the circadian rhythms of FT3 and TSH reflects this example of physiological control (75). *In vitro* and *in vivo* studies on athyreotic patients under levothyroxine (LT4) treatment further suggested a direct role of TSH in integrating cooperative elements of central and peripheral control (27, 76–80). Recent pathophysiological support from studies in the rat or genetically modified animals suggests that defending appropriate FT3 concentrations has high priority (81–83). As reviewed elsewhere (84, 85), invasive procedures carried out in the animals, not ethically possible in humans, extend many of our findings to tissue equilibria.

The newly proposed concept of relational stability assigns maintenance of T3 stability equal physiological relevance to central set point control. Where conflicts between the two regulatory elements may arise, T3 stability takes priority over set point maintenance. Importantly, this indicates that the set point is not only dramatically adjusted in extreme conditions such as the non-thyroidal illness syndrome, as has long been recognized (86, 87), but may be modified as part of an early response of the system when challenged by minor disturbances.

Understanding the progressive variation in control responses has important implications for clinical decision-making. First, it brings a homeostatic perspective to the controversial debate on the validity of TSH reference limits. Lowering the conventional reference range for TSH to 2 mIU/l has been proposed by some authors, based on imposing a statistical normal distribution, so as to sensitively define subclinical hypothyroidism (40, 45). Others see no need to redefine the upper reference limit (39, 40). If the non-random statistical pattern is explained by subtle heterogeneity in the expression of control in euthyroid individuals, the 2 mIU/l limit may not necessarily reflect the beginning of a diseased state, rather indicating a compensatory response to early capacity stress. This favors a more conservative approach for treatment decisions in patients with subclinical hypothyroidism (32, 70). However, given considerable individual variation, such findings of modest elevations in TSH should not countervail appropriate treatment if clinical presentation warrants it. Second, some unexpected outcomes may arise from balancing effects between the feedback and feedforward regulation. LT4 administration for instance may impair feedforward regulation by reducing stimulatory TSH levels more than anticipated from the added supply of exogenous T4, resulting in decreased FT3 concentrations in patients with autoimmune thyroiditis (88). This encourages further clinical study of calculated homeostatic parameters and interrelational measures (89).

## Interrelational Measures and Emerging New Concepts of Thyroid Homeostasis

While TSH alone can play a role as a sensitive screening test in asymptomatic subjects, it cannot also simultaneously assume the second role of a reliable diagnostic tool (gold standard) for defining true euthyroidism. The clinical interpretation of TSH should therefore be appropriately scaled back. TSH reference intervals for euthyroidism are uncertainly defined. From a homeostatic perspective, all three parameters TSH, FT4, and FT3 must be viewed together (27, 88).

To develop this concept further, we propose archival markers should be laid down for the individual subject. This may serve as a homeostatic reference point if any disturbance or thyroid disease arises in the future. Such markers will be readily available prior to surgery and may deliver personal targets to guide dose titration. In situations where they are unavailable or not applicable (e.g., after surgery for toxic adenoma or toxic multinodular goiter), reconstructing the set point from multiple TSH–FT4 pairs may offer an alternative option (90–92). As a strength, the latter approach provides individual markers with less variation, compared to univariate or multivariate reference ranges, but it has the disadvantage of failing to deliver FT3 targets and being sensitive to variations in deiodinase activity.

Potential treatment-related adjustments are not considered by this method, and the path of set points from hypothyroidism to euthyroidism can usually only be mapped in individuals under open loop conditions, i.e., under LT4 therapy in most cases. This becomes clinically relevant, because the set point has been demonstrated to differ in the same patient from health to disease, e.g., before and after thyroidectomy (27, 88, 93, 94). Hence, the equilibria in individuals appropriate for their healthy state do not remain unaltered and cannot act as equivalent targets for their diseased state. Consequently, log-linearity of the TSH–FT4 relationship cannot be safely assumed over the entire functional range (27, 95–99). While linearity roughly holds true in the open loop situation and hypothyroid state, the relationship becomes progressively damped toward the euthyroid range (88, 95, 99–101). Within the euthyroid range, factors other than TSH dominate the expression of control (27). Thereby, this fine-tunes the response, adjusting it to the conditional and situational needs. While this has been convincingly shown for populations in cross-sectional studies, longitudinal data are more difficult to interpret, because most patients followed are treated, and LT4-treatment has in itself a dose-related influence on the relationships (27, 88, 101).

**Figure 1:**
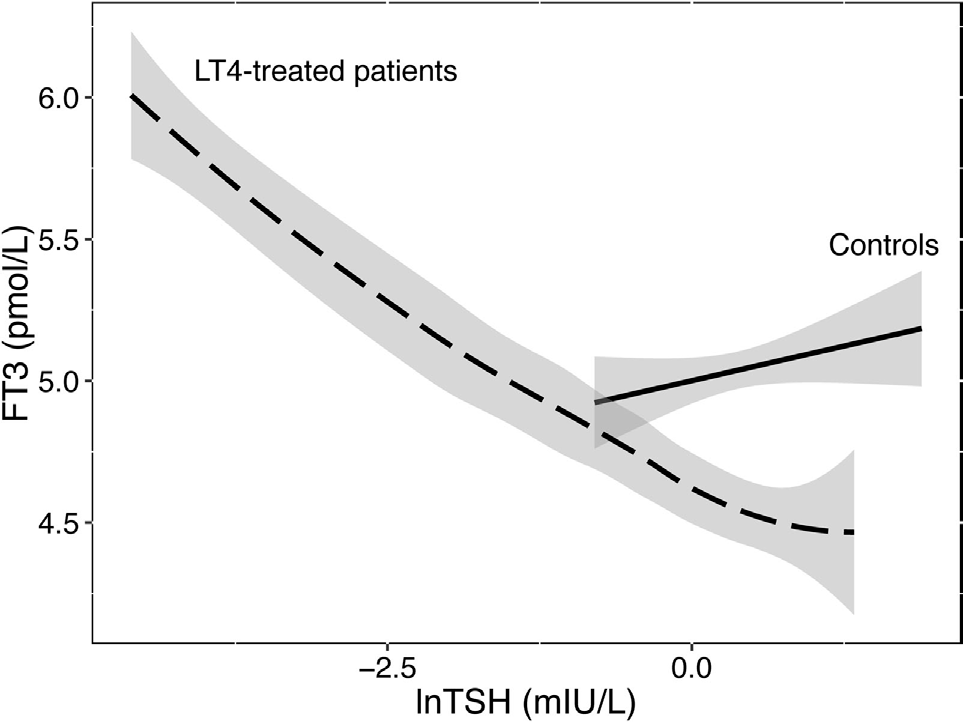
**Relationship between FT3 and TSH in healthy controls and LT4-treated patients**. While stable and uncorrelated with logarithmic TSH levels in controls, serum FT3 concentrations were unstably associated with TSH in LT4-treated patients. Data are from a published trial (88). The regression lines shown and their 95% confidence interval (shaded area) were fitted by a linear model in 207 controls or locally weighted scatterplot smoothing in 353 patients on LT4.

Athyreotic patients show particularly wide variations in their biochemical responses to LT4 treatment (102). This provokes alterations in the interlocking relationships and governing equilibria (27). While FT3 is uncorrelated with TSH in healthy subjects indicating T3 stability, it becomes TSH-related and unstable in LT4-treated patients (Figure 1). This flexible behavior precludes both the isolated interpretation of TSH levels and challenges the validity of the clinical application of fixed set points. Rather the balanced interrelationship between FT3 and TSH must be considered as a key component of control and should be interpreted more dynamically. FT3 as the primary target of stability cannot thus left be out of the equation. Neither can the importance of its absolute position be ignored nor can its equally important indirect influence on the TSH–FT4 relationship be neglected. However, equilibrium and optimum reference points may dissociate on LT4. Ingestion of a T4 load unaccompanied by the production of a physiologically appropriate T3-fraction may drive the system attempting regulatory balance into non-optimum positions (101). While LT4 dose escalation or the use of liquid formulations effectively control elevated TSH levels in various conditions including gastric problems, intestinal malabsorption, or drug interference (103, 104), this situation is different, as low FT3 concentrations persist in these patients despite suppressed TSH levels and elevated FT4 concentrations (102). Apparently, a minority of patients on LT4 still fail to concomitantly raise their serum FT3 concentrations as the T4 excess itself impairs T3–T4 conversion (102, 105). The clinical relevance was recently confirmed as euthyroid TSH targets could not adequately raise resting energy expenditure (REE) adjusted for lean body mass in LT4-treated women, compared to healthy controls, only FT3 levels being positively correlated with REE (106). A recent study using currently available evidence-based treatment options suggests hypothyroid patients can expect their quality of life to improve, but not a full recovery to a level characteristic of the healthy population (107). Recognizing and targeting homeostatic equilibria may improve future treatment strategies for hypothyroid patients, which remains an important goal.

## Conclusion

Thyroid-stimulating hormone gains unique properties from its embedment into a biological system and its primary role as a controlling element. Important differences thus arise exceeding the statistical concept of univariate normality conventionally applied to univariate reference ranges. Biological variation, homeostatic interrelations, equilibria, and set points between TSH, FT4, and FT3 become relevant for the observed expression of multivariate normality and relational stability in thyroid health. FT3 becomes a primary target for system stability whose absolute position and indirect influence on the TSH–FT4 relationship must be recognized. System instability may produce non-optimal equilibria under LT4 monotherapy in some patients. The triple roles of TSH as a sensitive screening test, an accurate diagnostic tool, and a therapeutic target require separation. A conjoint view of all thyroid parameters leads to the proposition of archival thyroid markers as future reference points.

## Author Contributions

RH, JM, and JD drafted the manuscript; RL contributed some revisions. All the authors read, amended, and approved the final manuscript.

## Conflict of Interest Statement

JD received funding and personal fees by Sanofi-Henning, Hexal AG and Pfizer, and is co-owner of the intellectual property rights for the patent “System and Method for Deriving Parameters for Homeostatic Feedback Control of an Individual” (Singapore Institute for Clinical Sciences, Biomedical Sciences Institutes, Application Number 201208940-5, WIPO number WO/2014/088516). All the other authors declare that there is no conflict of interest that could be perceived as prejudicing the impartiality of the research reported.
